# The communicative advantage: how kinematic signaling supports semantic comprehension

**DOI:** 10.1007/s00426-019-01198-y

**Published:** 2019-05-11

**Authors:** James P. Trujillo, Irina Simanova, Harold Bekkering, Asli Özyürek

**Affiliations:** 1 Donders Institute for Brain, Cognition and Behaviour, Radboud University, Montessorilaan 3, B.01.25, 6525GR Nijmegen, The Netherlands; 2 Centre for Language Studies, Radboud University, Nijmegen, The Netherlands; 3 Max Planck Institute for Psycholinguistics, Wundtlaan 1, 6525XD Nijmegen, The Netherlands

## Abstract

Humans are unique in their ability to communicate information through representational gestures which visually simulate an action (eg. moving hands as if opening a jar). Previous research indicates that the intention to communicate modulates the kinematics (e.g., velocity, size) of such gestures. If and how this modulation influences addressees’ comprehension of gestures have not been investigated. Here we ask whether communicative kinematic modulation enhances semantic comprehension (i.e., identification) of gestures. We additionally investigate whether any comprehension advantage is due to enhanced early identification or late identification. Participants (*n* = 20) watched videos of representational gestures produced in a more- (*n* = 60) or less-communicative (*n* = 60) context and performed a forced-choice recognition task. We tested the isolated role of kinematics by removing visibility of actor’s faces in Experiment I, and by reducing the stimuli to stick-light figures in Experiment II. Three video lengths were used to disentangle early identification from late identification. Accuracy and response time quantified main effects. Kinematic modulation was tested for correlations with task performance. We found higher gesture identification performance in more- compared to less-communicative gestures. However, early identification was only enhanced within a full visual context, while late identification occurred even when viewing isolated kinematics. Additionally, temporally segmented acts with more post-stroke holds were associated with higher accuracy. Our results demonstrate that communicative signaling, interacting with other visual cues, generally supports gesture identification, while kinematic modulation specifically enhances late identification in the absence of other cues. Results provide insights into mutual understanding processes as well as creating artificial communicative agents.

**Electronic supplementary material:**

The online version of this article (10.1007/s00426-019-01198-y) contains supplementary material, which is available to authorized users.

## Introduction

Human communication is multimodal, utilizing various signals to convey meaning and interact with others. Indeed, humans may be uniquely adapted for knowledge transfer, with the ability to signal the intention to interact as well as to manifest the knowledge that s/he wishes to communicate (Csibra & Gergely, 2006). This communicative signaling system is powerful in that the signals are dynamically adapted for the context in which they are used. For example, representational gestures (Kendon, 2004; McNeill, 1994) show systematic modulations dependent upon the communicative or social context in which they occur (Campisi & Özyürek, 2013; Galati & Galati, 2015; Gerwing & Bavelas, 2004; Holler & Beattie, 2005). Although these gestures are an important aspect of human communication, it is currently unclear how the addressee benefits from this communicative modulation. The current study aims to investigate for the first time whether and how kinematic signaling enhances identification of representational gestures.

There is growing evidence that adults modulate their action and gesture kinematics when communicating with other adults, depending on the communicative context. For example, adults adapt to addressees’ knowledge by producing gestures that are larger (Bavelas, Gerwing, Sutton, & Prevost, 2008; Campisi & Özyürek, 2013), more complex (Gerwing & Bavelas, 2004; Holler & Beattie, 2005), and higher in space (Hilliard & Cook, 2016) when conveying novel information. Instrumental actions intended to teach show similar kinematic modulation, including spatial (McEllin, Knoblich, & Sebanz, 2018; Vesper & Richardson, 2014) and temporal (McEllin et al., 2018) exaggeration. Evidence from our own lab corroborates these findings of spatial and temporal modulation in the production of both actions and gestures. In our recent work, we quantified the spatial and temporal modulation of actions and pantomime gestures (used without speech) in a more- relative to a less-communicative context (Trujillo, Simanova, Bekkering, & Özyürek, 2018). We showed that spatial and temporal features of actions and pantomime gestures are adapted to the communicative context in which they are produced.

A computational account by Pezzulo, Donnarumma, and Dindo (2013) suggests that modulation makes meaningful acts communicative by disambiguating the relevant information, effectively making the intended movement goal clear to the observer. This framework focuses on actions, but could be extended to gestures. One recent experimental study directly assessed how kinematic modulation affects gesture comprehension. By combining computationally based robotic production of gestures with validation through human comprehension experiments, Holladay, Dragan, and Srinivasa (2014) showed that spatial exaggeration of kinematics allows observers to more easily recognize the target of pointing gestures. Similarly, Gielniak and Thomaz (2012) showed that when robot co-speech gestures are kinematically exaggerated, the content of an interaction with that robot is better remembered. Another study used an action-based leader–follower task to show that task leaders not only systematically modulate task-relevant kinematic parameters, but these modulations are linked to better performance of the followers (Vesper, Schmitz, & Knoblich, 2017).

These previous studies suggest that the kinematics modulation of communicative movements (e.g., actions and gestures) serves to clarify relevant information for the addressee. However, it remains unclear whether this also holds for more complex human movements, such as pantomime gestures. This question is important for our understanding of human communication given that complex representations form an important part of the communicative message (Kelly, Ozyurek, & Maris, 2010; Özyürek, 2014).

The mechanism by which kinematic modulation might support semantic comprehension, or identification, of complex movements remains unclear. Several studies suggest disambiguation of the ongoing act, either through temporal segmentation of relevant parts (Blokpoel et al., 2012; Brand, Baldwin, & Ashburn, 2002), or spatial exaggeration of relevant features (Brand et al., 2002) as the mechanism. In the case of disambiguation, the “semantic core” (Kendon, 1986), or meaningful part of the movement, is made easier to understand as it unfolds. However, there is also evidence suggesting that early kinematic cues provide sufficient information to inform accurate prediction of whole actions before they are seen in their entirety (Cavallo, Koul, Ansuini, Capozzi, & Becchio, 2016; Manera, Becchio, Cavallo, Sartori, & Castiello, 2011). One study, for example, used videos of a person walking, and at a pause in the video participants were asked whether the actress in the video would continue to walk, or start to crawl. The authors showed that whole-body kinematics could support predictions about the outcome of an ongoing action (Stapel, Hunnius, & Bekkering, 2012). However, another study showed videos of a person reaching out and grasping a bottle, and asked the participants to predict the next sequence in the action (e.g., to drink, to move, to offer) and found that they were unable to use such early cues for accurate identification in this more complex, open-ended situation (Naish, Reader, Houston-Price, Bremner, & Holmes, 2013). Furthermore, identification of pantomime gestures has previously been reported to be quite low when no contextual (i.e., object) information is provided (Osiurak, Jarry, Baltenneck, Boudin, & Le Gall, 2012). Given these inconsistencies in the literature, an open question remains: are early kinematic cues sufficient to inform early representational gesture identification, or does kinematic modulation primarily aid gesture identification as the movements unfold (i.e., late identification)?

Finally, to understand how kinematic modulation might support gesture identification, it is important to consider other factors that might influence the semantic comprehension of an observer. In a natural environment, movements such as gestures are accompanied by additional communicative signals, such as facial expression and eye-gaze, and/or finger kinematics relevant in the execution of the gestures. Humans are particularly sensitive to the presence of human faces, which naturally draw attention (Cerf, Harel, Einhäuser, & Koch, 2007; Hershler & Hochstein, 2005; Theeuwes & Van der Stigchel, 2006). This effect is most prominent in the presence of mutual gaze (Farroni, Csibra, Simion, & Johnson, 2002; Holler et al., 2015), but also occurs in averted gaze compared to non-face objects (Hershler & Hochstein, 2005). Hand-shape information can also provide clues as to the object one is manipulating (Ansuini et al., 2016), and more generally the kinematics of the hand and fingers together provide early cues to upcoming actions (Becchio, Koul, Ansuini, Bertone, & Cavallo, 2018; Cavallo et al., 2016), which together may allow the act to be more easily identified. To understand the role of kinematic modulation in communication, the complexity of the visual scene must also be taken into account.

In sum, previous studies show kinematic modulation occurring as a communicative cue in actions and gestures. While research suggests that this modulation serves to enhance comprehension, this has not been assessed directly in terms of semantic comprehension of complex movements, such as representational gestures. Furthermore, it is currently unclear if improved comprehension would be driven by early action identification or by late identification of semantics, and which kinematic features provide this advantage.

The current study addresses these questions. In two experiments, naïve participants perform a recognition task of naturalistic pantomime gestures recorded in our previous study (Trujillo, Simanova et al., 2018). In the first experiment, they see the original videos with the face of the actor either visible or blurred, to control for eye-gaze effects. In the second experiment, the same videos are reduced to stick-light figures, reconstructed from Kinect motion tracking data. The stick figure videos allow us to test the contribution of specific kinematic features, because only the movements are visible, but not the face or hand shape. In both experiments, we additionally manipulate video length to test whether any communicative benefit is driven more by early identification (resulting in differences only in the initial fragment), or late identification (resulting in differences in the medium and full fragments). Experiment II provides an additional exploratory test of the contribution of specific kinematic features to gesture identification.

We hypothesize that kinematic modulation serves to enhance semantic legibility. As early kinematic information is less reliable for open-ended action prediction (Naish et al., 2013) and pantomime gestures may generally be difficult to identify without context (Osiurak et al., 2012), we expect better recognition scores for the communicative gestures in the medium fragments and full fragments compared to initial fragments. We furthermore predict that performance will correlate with stronger kinematic modulation. Additionally, we expect performance to be lower overall with stick-light figures, compared to the full videos due to decreased visual information, but with a similar pattern (i.e., better performance in medium and full fragments compared to initial). For our exploratory test, we expect that exaggeration of both spatial and temporal kinematic features will contribute to better gesture identification.

## Experiment I: Full visual context

Our first experiment, with actual videos of the gestures, was designed to test whether (1) kinematic modulations lead to improved semantic comprehension in an addressee, (2) if the advantage is better explained by early identification or late identification of the gestures, and (3) whether the effect is altered by removing a salient part of the visual context, the actor’s face.

### Methods

#### Participants

Twenty participants were included in this study (mean age = 28; 16 female), recruited from the Radboud University. Participants were selected on the criteria of being aged 18–35, right-handed and fluent in the Dutch language, with no history of psychiatric disorders or communication impairments. The procedure was approved by a local ethics committee and informed consent was obtained from all individual participants in this study.

#### Materials

Each participant performed the recognition task with 60 videos of pantomimes that differed in their context (more or less communicative), video duration (short, medium and full), and face visibility (face visible vs. blurred). Detailed description of the video recordings, selection and manipulation follows below.

##### Video recording procedure

Stimuli were derived from a previous experiment (Trujillo, Simanova et al., 2018). In this previous experiment, participants (henceforth, actors) were filmed while seated at a table, with a camera hanging in front of the table. Motion-tracking data were acquired using Microsoft Kinect system hanging slightly to the left of the camera. Each actor performed a set of 31 gestures, either in a more-communicative or a less-communicative setting (described below). Gestures consisted of simple object-directed acts, such as cutting paper with scissors or pouring water into a cup. Target objects were placed on the table (e.g., scissors and a sheet of paper for the item ‘cut the paper with the scissors’) but actors were instructed to perform as if they were acting on the objects, without actually touching them. For each item, actors began with their hands placed on designated starting points on the table (marked with tape). After placing the target object(s) on the table, the experimenter moved out of view from the participant and the camera, and recorded instructions were played. Immediately following the instructions, a bell sound was played, which indicated that the participant could begin with the pantomime. Once the act was completed, actors returned their hands to the indicated starting points, which elicited another bell sound, and waited for the next item. For this study, videos began at the first bell sound, and ended at the second bell sounded. In the more-communicative context we introduced a confederate who sat in an adjacent room and was said to be watching through the video camera and learning the gestures from the participant. In this way, an implied communicative context was created. In the less-communicative context, the same confederate was said to be learning the experimental setup. The less-communicative context was, therefore, exactly matched, including the presence of an observer, but only differed in that there was no implied interaction. Despite the subtle task manipulation, our previous study (Trujillo, Simanova et al., 2018) showed robust differences in kinematics between the gestures produced in the more-communicative vs. the less-communicative context.

#### Kinematic feature quantification

For the current study, we used the same kinematic features that were quantified in our earlier study (Trujillo, Simanova et al., 2018). We used a toolkit for markerless automatic analysis of kinematic features, developed earlier in our group (Trujillo, Vaitonyte, Simanova, & Özyürek, 2018). The following briefly describes the feature quantification procedure: all features were measured within the time frame between the beginning and the ending bell sound. Motion-tracking data from the Kinect provided measures for our kinematic features, and all raw motion-tracking data were smoothed using the Savitzky–Golay filter with a span of 15 and degree of 5. As described in our previous work (Trujillo, Simanova et al., 2018), this smoothing protocol was used as it brought the Kinect data closely in line with simultaneously recorded optical motion-tracking data in a separate pilot session. The following features were calculated from the smoothed data: *Distance* was calculated as the total distance traveled by both hands in 3D space over the course of the item. *Vertical amplitude* was calculated on the basis of the highest space used by either hand in relation to the body. *Peak velocity* was calculated as the greatest velocity achieved with the right (dominant) hand. *Hold time* was calculated as the total time, in seconds, counting as a hold. Holds were defined as an event in which both hands and arms are still for at least 0.3 s. *Submovements* were calculated as the number of individual ballistic movements made, per hand, throughout the item. To account for the inherent differences in the kinematics of the various items performed, *z* scores were calculated for each feature/item combination across all actors including both conditions. This standardized score represents the modulation of that feature, as it quantifies how much greater or smaller the feature was when compared to the average of that feature across all of the actors. (Addressee-directed) Eye-gaze was coded in ELAN as the proportion of the total duration of the video in which the participant is looking directly into the camera. For a more detailed description of these quantifications, see Trujillo, Simanova et al. (2018). Also note that the kinematic features calculated using this protocol are in line with the same features manually annotated from the video recordings (Trujillo, Vaitonyte et al., 2018). This supports our assumption that the features calculated from the motion-tracking data represent qualities that are visible in the videos.

##### Inclusion and randomization

Our stimuli set included 120 videos (of the 2480) recorded in our previous study (Trujillo, Simanova et al., 2018). Our selection procedure (see Appendix 1) ensured that our stimulus set in the present experiment included an equal number of more- and less-communicative videos. Each of the 31 gesture items from the original set was included a minimum of three times and maximum of four times across the entire selection, performed by different actors, while ensuring that each item also appeared at least once in the more-communicative context and once in the less-communicative context. Three videos from each actor in the previous study were included. Appendix 2 provides the full list of items gesture items. Supplementary Figure 1 illustrates the range of kinematics, gaze, and video durations included across the two groups in the current study with respect to the original dataset from Trujillo, Simanova et al. (2018). We ensured that the stimulus set for the present study matched the original dataset in terms of context-specific differences in the kinematics and eye-gaze, ensuring that the current stimulus set is a representative sample of the data shown in Trujillo, Simanova et al. (2018). These results are provided in Appendix 1.

#### Video segmentation

**Fig. 1:**
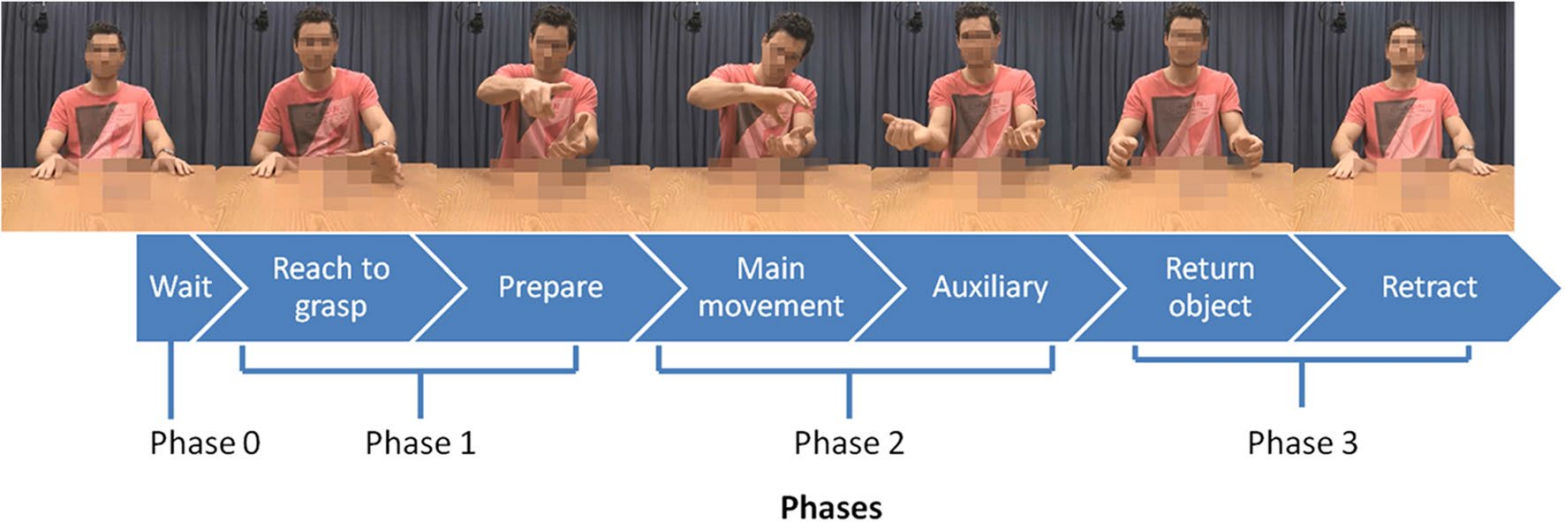
Overview of video segmentation and phases. Along the top, representative still frames are shown throughout one video (item: “open jar”). The individual blue blocks indicate individual segments. Below this, phase division is depicted (color figure online)

To test whether kinematic modulation primarily influences early or late identification (question 2), we divided the videos into segments of different length. Based on the previous literature (Kendon, 1986; Kita, van Gijn, & van der Hulst, 1998), we defined segments as following: *Wait* covered the approximate 500 ms after the bell was played, but before the participant started to move. *Reach to grasp* covered the time during which the participant reached towards, and subsequently grasped the target object. In the case of multiple objects, this segment ended after both objects were grasped. *Prepare* captured any movements unrelated to the initial reach to grasp, but was not part of the main semantic aspect of the pantomime. *Main movement* covered any movements directly related to the semantic core of the item. *Auxiliary* captured any additional movements not directly related to the semantic core. *Return object* captured the movement of the hands back to the objects starting position, depicting the object being replaced to its original location. *Retract* covered the movement of the hands back to the indicated the starting position of the hands, until the end of the video. Note that the “prepare”, and “auxiliary” segments were optional, and only coded when such movements were present. All other segments were present in all videos. Phases were delineated based on this segmentation. *Phase 0* covered the “wait” segment. *Phase 1* covered “reach to grasp” and “prepare”. *Phase 2* covered the “main movement” and “auxiliary”. *Phase 3* covered “return object” and “retract”. See Table 1 and Fig. 1 for examples of how these phases map onto specific parts of the movement.

**Table 1:** Movement phase examples

	Phase 1		Phase 2		Phase 3	
	Reach-to-grasp	Prepare	Main movement	Auxiliary	Return object	Retract
Open jar	Right hands extends to jar	Right hand lifts jar. Left hand grasps lid	Twisting hands to depict unscrewing the lid	Hands moved apart to show separating lid from jar	Hands return to object starting positions	Hands returned to indicated starting position
Cut paper	Right hand extends to scissors, left hand to paper	Both hands lifted, configured to start cutting paper	Cutting motion depicted with right hand	Hands spread apart to show that the cutting is complete	Hands return to object starting positions	Hands returned to indicated starting position

After defining the segments for each video, we divided the videos into three lengths, referred to as initial fragments (*M* = 3.27 ± 1.52 s), medium fragments (*M* = 4.62 ± 2.19 s), and full videos (*M* = 5.59 ± 2.53 s). Initial fragments consisted of only phase 0 and phase 1, medium fragments consisted of phases 0–2, and full videos contained all of the phases. An overview of these segments and phases can be seen in Fig. 1. We performed ANOVAs on each of the fragment lengths to ensure video durations of the same fragment length did not differ significantly across cells (see Supplementary Table 1 for statistics). This resulted in initial fragments only providing initial hand-shape and arm/hand/finger configuration information, medium fragments providing all relevant semantic information, and full videos providing additional eye-gaze (when present) and additional time for processing the information.

##### Blurring

In all videos, a Gaussian blur was applied to the object, which was otherwise visible in the video. This ensured that the object could not be used to infer the action. To determine whether the face in general, in particular the gaze direction, has an effect on pantomime recognition, we also applied a Gaussian blur to the face in half of the videos. Blurring the faces in this way allowed us to manipulate the amount of available visual information, providing a first test for how kinematic modulation affects gesture identification in a less complete visual context (question 3). This was balanced so that each actor had at least one video with a visible face and one with a blurred face.

#### Task

Before beginning the experiment, participants received a brief description of the task to inform them of the nature of the stimuli. This ensured that the participants knew to expect incomplete videos in some trials. Participants were seated in front of a 24″ Benq XL2420Z monitor with a standard keyboard for responses. Stimuli were presented at a frame rate of 29 frames per second, with a display size of 1280 × 720. During the experiment, participants would first see a fixation cross for a period 1000 ms with a jitter of 250 ms. One of the item videos was then displayed on the screen, after which the question appeared: “What was the action being depicted?” Two possible answers were presented on the screen, one on the left, and one on the right. Answers consisted of one verb and one noun that captured the action (e.g., the correct answer to the item “pour the water into the cup” was “pour water”). Correct answers were randomly assigned to one of the two sides. The second option was always one of the possible answers from the total set. Therefore, all options were presented equally often as the correct answer and as the wrong (distractor) option. Participants could respond with the 0 (left option) or 1 (right option) keys on the keyboard. Accuracy and response time (RT) were recorded for each video.

#### Analysis

##### Main effects analyses: communicative context, fragment length, and visual context

Both RT and accuracy of identification judgments were calculated for each of 12 cells (Table 2): fragment length (initial fragment vs. medium fragment vs. full video) × face (blurred vs. visible) × context (more-communicative vs. less-communicative) in order to test (1) whether more-communicative gestures were identified faster or with higher accuracy (main effect of context), (2) performance was higher in only initial fragments (providing evidence for early identification theory) or only in medium fragments (providing evidence for late identification), as well as (3) whether face visibility impacted performance, which informs us whether there is an effect of visual information availability on the identification performance. Separate repeated-measures analyses of variance (RM-ANOVA) were run for accuracy and RT to test for the presence of main and interactional effects. We used Mauchly’s test of sphericity on each factor and interaction in our model and applied the Greenhouse–Geisser correction where appropriate.

**Table 2:** Overview of analysis cells for Experiment I

	Context			
	Face visibility		Face visibility	
Fragment length	More-communicative Face visible Initial fragment Mean duration = 4.49	More-communicative Face blurred Initial fragment Mean duration = 5.03	Less-communicative Face visible Initial fragment Mean duration = 4.50	Less-communicative Face blurred Initial fragment Mean duration = 4.03
	More-communicative Face visible Medium fragment Mean duration = 4.72	More-communicative Face blurred Medium fragment Mean duration = 4.43	Less-communicative Face visible Medium fragment Mean duration = 4.34	Less-communicative Face blurred Medium fragment Mean duration = 4.57
	More-communicative Face visible Full fragment Mean duration = 4.73	More-communicative Face blurred Full fragment Mean duration = 4.34	Less-communicative Face visible Full fragment Mean duration = 4.29	Less-communicative Face blurred Full fragment Mean duration = 4.61

There are ten videos in each of the cells

### Results: Experiment I

**Fig. 2:**
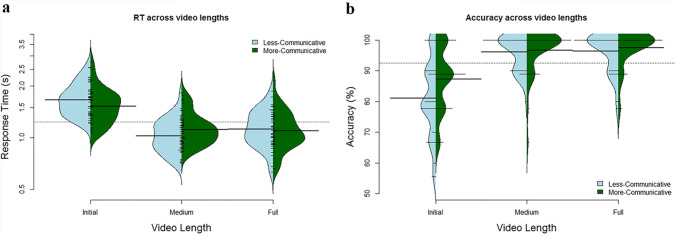
Overview of semantic judgment performance over context and fragment length, combined for face visibility. Bean plots depict the distribution (kernel density estimation) of the data. The dotted lines indicate the overall performance mean, the larger solid bars indicate the mean per video length and communicative context, shorter bars indicate mean values per participant, and the filled curve depicts the overall distribution of scores. Panel **a** shows mean accuracy across the three video lengths. Panel **b** shows RT across the three video lengths. In all panels, fragment length is depicted along the *x*-axis, the *y*-axis shows mean performance (in panel, mean accuracy; in panel, mean RT in seconds), while blue (left) plots depict the less-communicative context and green (right) plots the more-communicative context (color figure online)

We used RM-ANOVA to test for a significant main effect of communicative context, fragment length, or face visibility on performance. In terms of accuracy, results of the fragment length x face visibility x communicative context RM-ANOVA showed a significant main effect of communicative context, *F*(1,19) = 2.912, *p *= 0.029, as well as a main effect of fragment length, *F*(2,38) = 53.583, *p *< 0.001, but no main effect of face visibility, *F*(1,19) = 0.050, *p *= 0.825. Planned comparisons revealed higher accuracy in the more-communicative context for initial fragments (more-communicative mean = 87.13%, less-communicative mean = 81.17%; *t*(18) = 3.025, *p *= 0.007), but there was no difference between contexts in the medium fragments (more-communicative context mean = 97.37%, less-communicative mean = 96.49%; *t*(18) = 0.785, *p *= 0.443) or full videos (more-communicative mean = 97.37%, less-communicative mean = 97.22%; *t*(18) = 0.128, *p *= 0.899). In sum, performance was high overall on more-communicative compared to less-communicative videos, with specifically more-communicative initial fragments showing higher performance than less-communicative initial fragments. Accuracy, regardless of communicative context, was additionally higher in medium and full fragments compared to initial. See Fig. 2a for an overview of these results.

In terms of RT, results of the fragment length x face x context RM-ANOVA revealed a significant main effect of communicative context, *F*(1,19) = 5.699, *p *= 0.028, and of fragment length, *F*(2,38) = 192.489, *p *< 0.001, but not of face visibility, *F*(1,19) = 3.725, *p *= 0.069. Planned contrasts revealed faster RT in more-communicative compared to less-communicative initial fragments (more-communicative mean = 1.446; less-communicative mean = 1.583 s), *t*(19) = 3.824, *p *= 0.001 but faster RT for less- compared to more-communicative medium fragments (more-communicative mean = 1.094 s; less-communicative mean = 1.029 s), *t*(19) = 3.479, *p *= 0.003, but no difference between more- and less-communicative full videos (more-communicative mean = 1.094; less-communicative mean = 1.129), *t*(19) = 1.237, *p *= 0.231. We also found faster RT for medium fragments (*M* = 1.093) compared to initial fragments (*M* = 1.630), *t*(19) = 12.538, *p *< 0.001, as well as for medium fragments compared to full videos (*M* = 1.142), *t*(19) = 2.326, *p *= 0.031. In sum, RT was similar in both the more- and less-communicative contexts, but faster responses were seen in medium fragments compared to initial and full fragments. See Fig. 2b for an overview of these results.

### Discussion: Experiment I

In our first experiment, we sought to determine how communicative modulation affects identification of pantomime gesture semantics. We found that pantomime gestures produced in a more-communicative context were better recognized when compared to those produced in a less-communicative context. Specifically, more-communicative initial fragments were recognized more accurately and faster than less-communicative initial fragments.

The higher accuracy in recognizing more- compared to less-communicative initial fragments suggests that at least some of the relevant information is available even in the earliest stages of the act, and that communicative modulation enhances this information. Since the face visibility did not contribute significantly to better performance, we suggest that improved comprehension may come from fine-grained kinematic cues, such as hand-shape and finger kinematics. As objects are known to have specific action and hand-shape affordances (Grèzes & Decety, 2002; Tucker & Ellis, 2001), hand shape can also provide clues as to the object being grasped, and thus also the upcoming action (Ansuini et al., 2016; van Elk, van Schie, & Bekkering, 2014). These results are therefore in line with the early prediction results described for action chains (Becchio, Manera, Sartori, Cavallo, & Castiello, 2012; Cavallo et al., 2016). Our results may also be explained by immediate comprehension. In other words, the visual information provided by the shape and configuration of the hands may be sufficiently clear to activate the semantic representation of the action without any prediction of the upcoming movements. Although we cannot determine the exact cognitive mechanism, we can conclude that communicative modulation supports comprehension through early action identification.

We found no evidence for higher accuracy in more- compared to less-communicative medium fragments, nor for full videos. It seems that the overall accuracy in medium and full fragments does not allow a difference to be found between the contexts. In both more- and less-communicative medium fragments, accuracy was above 96%, suggesting that ceiling level performance may have already been reached. This indicates that even if communicative modulation supports late identification, general task difficulty was not high enough in our task to allow us to find any difference. Surprisingly, faster RT was found for less- compared to more-communicative medium fragments. This unexpected result may reflect a trade-off between kinematic modulation, which is thought to be informative, and direct eye-gaze, which serves a communicative function but may not lead to faster responses. Along this line, Holler and colleagues (2012) argue that direct eye-gaze leads to a feeling of being addressed, which in turn forces the addressee to split their attention between the eyes and hands of the speaker. If this interpretation is correct, we would expect that although responses are faster for the less-communicative videos, accuracy should still be higher in the more-communicative videos. To draw any conclusions about how communicative modulation affects late identification, we suggest that it is necessary to increase task difficulty.

In sum, our results show that communicatively produced gestures are more easily recognized than less communicative gestures, and that this effect is explained by early action identification. This result is in line with the research on child-directed actions (Brand et al., 2002), as well as the more recent developments regarding early action identification based on kinematic cues (Ansuini, Cavallo, Bertone, & Becchio, 2014; Cavallo et al., 2016).

## Experiment II: Isolated kinematic context

Although this first experiment shows evidence for a supporting role of kinematic modulation in semantic comprehension of gestures, it remains unclear whether the effect remains when only gross kinematics are observed, and facial, including attentional cueing to the hands, and finger kinematics, including hand shape, are completely removed. Removing additional visual contextual information would therefore help to disentangle the effects of gross (i.e., posture and hands) kinematic modulation from other (potentially communicative) visual information. For example, while extensive research has looked at the early phase of action identification from hand and finger kinematics (Ansuini et al., 2016; Becchio et al., 2018; Cavallo et al., 2016), the higher level dynamics of the hands and arms, which we call gross kinematics, have not been well studied. This is particularly relevant as these high level kinematic features are similar to the qualities described in gesture research. Thus, in Experiment II we replicate Experiment I, but reduce the stimuli to present a visually simplistic scene consisting of only lines representing the limbs of the actor’s body. If kinematic modulation is driving the communicative advantage seen in our first experiment, we can expect the same effect pattern as seen in Experiment I. If other features of the visible scene, such as finger kinematics, provided the necessary cues for semantic comprehension then the effect on early identification should no longer be present. Due to the visual information being highly restricted, we expect task difficulty to be increased.

In this way, we are able to determine if kinematic modulation supports early action identification in the absence of other early cues such as hand shape, and whether it supports ongoing semantic disambiguation when gesture recognition is more difficult. Overall, this experiment will build on our findings from Experiment I by providing a specific test of how kinematic modulation affects semantic comprehension when isolated from other contextual information. Additionally, it will test which specific kinematic features contribute to supporting semantic comprehension.

### Methods: Experiment II

#### Participants

Twenty participants were included in this study (mean age = 24; 16 female), recruited from the Radboud University. Participants were selected on the criteria of being aged 18–35, right-handed, fluent in the Dutch language, without any history of psychiatric impairments or communication disorders, and not having participated in the previous experiment. The procedure was approved by a local ethics committee and informed consent was obtained from all individual participants in this study.

#### Materials

**Fig. 3:**
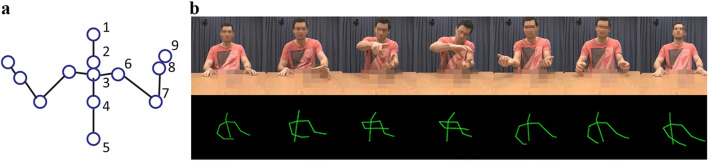
Illustration of materials used for Experiment II. **a** Diagram of joints represented in the videos of Experiment II: 1. top of head, 2. bottom of head, 3. top of spine, 4. middle of spine, 5. lower spine, 6. shoulder, 7. elbow, 8. wrist, 9. center of hand. Note that numbers 6–9 are present for both the left and right arms. **b** Still frames from an actual stimulus video, depicting the visual information made available to the participants, underneath the corresponding actual video frames (not shown to participants) for comparison

We used same video materials as in the Experiment I, but this time the videos were reduced to stick-light-figures. Motion-tracking data were used to reconstruct the movements of the upper-body joints (Trujillo, Vaitonyte et al., 2018). Videos consisted of these reconstructions, using *x*, *y*, *z* coordinates acquired at 30 frames per second of these joints (see Fig. 3 for an illustration of the joints utilized). Note that no joints pertaining to the fingers were visually represented. This ensured that hand shape was not a feature that could be identified by an observer. These points were depicted with lines drawn between the individual points to create a light stick figure, representing the participants’ kinematic skeleton. Skeletons were centered in space on the screen, with the viewing angle adjusted to reflect an azimuth of 20° and an elevation of 45° in reference to the center of the skeleton.

#### Analysis

##### Main effects analyses: communicative context, fragment length, and visual context

To determine if there was an overall effect of communicative context on accuracy or RT, and to again test for evidence of either the early identification or late identification hypothesis, we used two separate 3 (fragment length) × 2 (context) one-way ANOVAs. When appropriate, independent samples *t* tests were used to determine where these differences occurred across the three video lengths. When a non-normal distribution was detected, results are reported after a Greenhouse–Geisser correction.

##### Feature level regression analysis: exploratory test of kinematic modulation values

Given that Experiment II aims to test the specific contribution of kinematic modulation on semantic comprehension, we additionally performed an exploratory linear mixed effects analysis using the kinematic modulation values that characterize the stimulus videos. This was done to assess the relation between specific kinematic features and semantic judgment performance. Kinematic modulation values were available from our previous study, where these stimulus videos were created (Trujillo, Simanova et al., 2018), and were meant to quantify kinematic features in the semantic core of the action. We, therefore, chose to perform this additional analysis in Experiment II as a follow-up assessment of the significant difference between more- and less-communicative medium fragments (Table 3).

**Table 3:** Overview of analysis cells for Experiment II

	Context	
Fragment length	More-communicative Initial fragment Mean = 4.22 s	Less-communicative Initial fragment Mean = 4.24 s
	More-communicative Medium fragment Mean = 4.68 s	Less-communicative Medium fragment Mean = 4.73 s
	More-communicative Full fragment Mean = 4.59 s	Less-communicative Full fragment Mean = 4.51 s

There are ten videos in each of the cells

We performed linear regression analyses between the set of kinematic features and RT, and a logistic regression between the set of kinematic features and accuracy. Regression analyses were performed on the medium fragments, as this is where a statistically significant difference was found between more- and less-communicative videos. Statistical analyses utilized mixed effects models implemented in the R statistical program (R Core Team, 2014) using the lme4 package (Bates, Mächler, Bolker, & Walker, 2014). *p* values were estimated using the Satterthwaite approximation for denominator degrees of freedom, as implemented in the lmerTest package (Kuznetsova, 2016). Our regression models first factored out video duration and subsequently tested the three main components of kinematic modulation that have been identified in previous research: range of motion (Bavelas et al., 2008; Hilliard & Cook, 2016) (here quantified as vertical space utilized), velocity of movements, and punctuality (Brand et al., 2002) (here quantified as the number of submovements and the amount of holds between them. Kinematic features were defined as main effects, while a random intercept was added for participant. For a detailed description of how the model was defined, see Appendix 3. To reduce the risk of Type I error, we used the Simple Interactive Statistical Analysis tool (http://www.quantitativeskills.com/sisa/calculations/bonfer.htm) to calculate an adjusted alpha threshold based on the mean correlation between all of the tested features (regardless of whether they are in the final model or not), as well as the number of tests (i.e., number of variables remaining in the final mixed model). Our six variables (duration, vertical amplitude, peak velocity, submovements, hold time) showed an average correlation of 0.154, leading to a corrected threshold of *p *= 0.019.

### Results: Experiment II

#### Main effects analyses: communicative context, fragment length

Our first RM-ANOVA tested whether accuracy was affected by the communicative context, or the fragment length of the videos. We found a significant main effect of communicative context on accuracy, *F*(1,19) = 5.108, *p* = 0.036, as well as a main effect of fragment length, *F*(2,38) = 10.962, *p *< 0.001. Planned comparisons revealed no difference between accuracy of more-communicative and less-communicative initial fragments (more-communicative mean = 59.58%, less-communicative mean = 56.76%), *t(*19) = − 0.646, *p *= 0.526, or in full videos (more-communicative mean = 64.87%, less-communicative mean = 62.76%), *t*(19) = 0.492, *p *= 0.628. We found significantly higher accuracy in more-communicative medium fragments (*M* = 75.69%) compared to less-communicative medium fragments (*M* = 66.11%) videos, *t*(19) = 2.99, *p *= 0.007. We found no fragment length by communicative context interaction, *F*(2,36) = 0.659, *p* = 0.523.

**Fig. 4:**
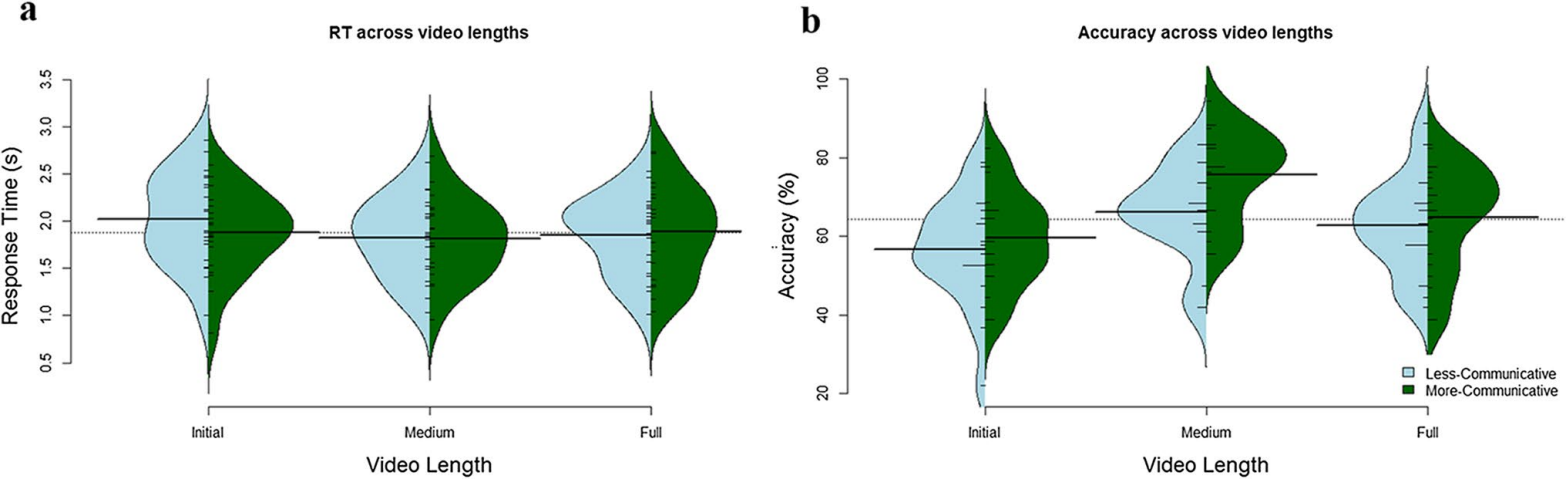
Overview of semantic judgment performance over context and fragment length in Experiment II. Bean plots depict the distribution (kernel density estimation) of the data. The dotted lines indicate the overall performance mean, the largest solid bars indicate the group mean per video length and context, and shorter bars indicate individual participant means. Panel **a** shows mean accuracy across the three video lengths. Panel **b** shows RT across the three video lengths. In all panels, fragment length is depicted along the *x*-axis, the *y*-axis shows mean performance (in panel, mean accuracy; in panel, mean RT in seconds), while blue (left) plots depict the less-communicative context and green (right) plots the more-communicative context (color figure online)

Our second RM-ANOVA tested whether RT was affected by communicative context or fragment length. We found a significant main effect of fragment length on RT, *F*(2,38) = 7.263, *p* = 0.003, but no main effect of communicative context, *F*(1,19) = 2.12, *p *= 0.162. We additionally found a video length x context interaction, *F*(2,38) = 3.87, *p *= 0.031. Planned comparisons revealed significantly faster RT in medium fragments (*M* = 1.817 s) compared to initial fragments (*M* = 1.953 s), *t*(19) = 3.982, *p* = 0.001, but no difference between medium fragments and full videos (*M* = 1.872 s), *t*(19) = 1.339, *p* = 0.196. See Fig. 4 for an overview of these results. In sum, communicative context did not affect RT, but responses were faster in medium compared to initial fragments.

#### Feature level regression analysis: exploratory test of kinematic modulation values

To test which specific kinematic features, if any, affected accuracy, we used mixed models to assess whether accuracy on each video could be explained by the kinematic features of that video. We found kinematic modulation of punctuality (hold-time and submovements) to explain performance accuracy better than the null model, *χ* ^2^(5) = 16.064, *p *< 0.001. Specifically, increased hold time was associated with higher accuracy (*b* = 0.377, *z* = 3.962, *p *< 0.001), although submovements were not (*z* = − 0.085, *p *= 0.932). We found no correlation between duration and accuracy (*z* = − 1.151, *p *= 0.249) in our kinematic model. Response time was not significantly explained by any of the kinematic feature sets. Duration, as assessed in the null model, was also not related to response time (*t* = − 1.768, *p *= 0.077). In sum, kinematic modulation of hold time was specifically related to higher performance accuracy.

### Discussion: Experiment II

Experiment II was designed to test the isolated contribution of kinematics to semantic comprehension and further differentiate between early identification vs. late identification. We found that more-communicative videos were still recognized with overall higher accuracy than less-communicative videos even in the absence of contextual cues such as hand-shape, finger kinematics, or actor’s face.

Higher accuracy in recognizing more-communicative compared to less-communicative medium fragments suggests that the advantage given by kinematic modulation predominantly affects identification of the pantomime after it has unfolded. The unfolding of the final phase of the pantomime may provide enough extra time for the overall act to be processed completely and the pantomime to be recognized accurately regardless of modulation. This finding is therefore in line with the hypothesis that kinematic modulation mainly contributes to ongoing semantic disambiguation. We further explored the contribution of specific kinematic features to semantic comprehension in the absence of further visual context such as hand shape or facial cues. We found that temporal kinematic modulation (i.e., increasing segmentation of the act) was an important factor influencing semantic comprehension. Specifically, increasing hold time positively impacted accuracy. Our results suggest that although the effect may be subtle in production, this feature plays an important role in clarifying semantic content through temporal unfolding of the gesture.

## General discussion

This study aimed to determine the role of kinematic modulation in the semantic comprehension of (pantomime) gestures. First, we asked whether kinematic modulation influences semantic comprehension of gestures and found that more-communicatively produced gestures are recognized better than less-communicatively produced gestures (Experiments I and II). Second, by utilizing different video fragment lengths, we tested the underlying mechanism of this communicative advantage. We found evidence for enhanced early identification when provided with a more complete visual scene, including the hand shape (Experiment I), but enhanced late identification when providing with only gross kinematics (Experiment II). Finally, we show in Experiment II that increased post-stroke hold time has the strongest effect on the communicative gesture comprehension advantage.

When provided with a wealth of visual cues, as in Experiment I, participants gained a communicative advantage even in the early stages of movement. This finding fits nicely with the idea that the end goal of an action, or perhaps the upcoming movements themselves, can be predicted by utilizing early kinematics together with visual contextual information (Cavallo et al., 2016; Iacoboni et al., 2005; Stapel et al., 2012). Our results from the Experiment II suggest that kinematic modulation of gross hand movements alone is not sufficient for this effect as when the visual stimulus was degraded this advantage was removed. It should be noted that we cannot conclude that kinematic information is insufficient, but rather that the gross hand kinematics that are typically used to assess gestures are insufficient. This is particularly relevant given the evidence that hand and finger kinematics inform early manual action identification (Becchio et al., 2018; Cavallo et al., 2016; Manera et al., 2011). We, therefore, conclude that both kinematic and non-kinematic cues play a role in early gesture recognition, while modulated arm and hand kinematics provide cues to identify the act as it unfolds, even in the absence of other visual cues.

Our conclusion regarding the role of temporal modulation, and more specifically the increased hold time, as supporting semantic comprehension matches well with the factor ‘punctuality’, as defined by Brand et al. (2002) in their study of child-directed action. Punctuality of actions refers to movement segments with clear beginning and end points, allowing the individual movements to be clear to an observer (Blokpoel et al., 2012). Exaggerating the velocity changes between movements and increasing hold time (Vesper et al., 2017) can make the final body configuration more salient by allowing longer viewing time of this configuration for the addressee.

Our findings have several important implications. By combining naturalistic motion-tracking production data with a semantic judgment task in naïve observers, our study provides new insights and support for models of effective human–machine interactions. Specifically, our results expand and contrast the robotics literature that demonstrate spatial modulation as a method of defining more legible acts (Dragan, Lee, & Srinivasa, 2013; Dragan & Srinivasa, 2014; Holladay et al., 2014). Our findings suggest that while spatial modulation may be effective for single-movement gestures such as pointing, temporal modulation has a larger role in this clarification effect in more complex acts.

We additionally build on studies of gesture comprehension, showing the importance of kinematic cues in successful semantic uptake and bringing new insights into previous findings. For instance, our findings provide a mechanistic understanding of larger scale, qualitative features, such as informativeness (Campisi & Özyürek, 2013). Differences in the informativeness of complex gestures may be understood by looking at the underlying kinematic differences and how these relate to the comprehension of such gestures. As an example, gestures are understood through the individual movements that comprise them, rather than static hand configurations (Kendon, 2004; McNeill, 1994). Increasing the number of clearly defined movements consequently increases the amount of visual information available to an observer, which could lead to the perception of increased informativeness.

Our work has further implications for clinical practice, where it can be applied to areas such as communication disorders. Research has shown that people with aphasia use gestures, including pantomimes, to supplement the semantic content of their speech (DeBeer et al., 2015; Rose, Mok, & Sekine, 2017). Knowledge of which features contribute to semantically recognizable gestures could, therefore, be applied to developing therapies for more effective pantomime use and understanding.

## Summary

Our study is the first to systematically test and provide a partial account of how the kinematic modulation that arises from a more-communicative context can support efficient identification of a manual act. We found that communicatively produced acts are more easily understood early on due to kinematic and non-kinematic cues. While comprehension is dependent on how much of the visual scene is available, communicative kinematic modulation alone leads to improved recognition of pantomime gestures even in a highly reduced visual scene. Particularly, temporal kinematic modulation leads to improved late identification of the act in the absence of other cues.

### Electronic supplementary material

Below is the link to the electronic supplementary material.

Supplementary material 1 (R 6 kb)

Supplementary material 2 (R 7 kb)

Supplementary material 3 (TXT 192 kb)

Supplementary material 4 (TXT 450 kb)

Supplementary material 5 (DOCX 1830 kb)

## Appendix 1: Item selection procedure

To provide a representative sampling of each of the two groups, all individual items from all subjects included in the previous study were ranked according to eye-gaze and overall kinematic modulation (i.e., *z* scores derived from the kinematic features described in the section *b*). The two groups were ordered such that items with high values for addressee-directed eye-gaze and kinematic modulation were ranked higher than those with low values. This placed all items on a continuum that ranked their communicativeness. This was done due to the observation that, due to the subtle manipulation of context in Experiment I of Trujillo, Simanova et al. (2018), there was considerable overlap of kinematic modulation in the middle of the spectrum (i.e., some actors in the more-communicative context showed modulation more similar to those of the less-communicative context, and vice versa). We chose to include items which represented a range of eye-gaze and kinematic features representative of their respective communicative context. This method allowed a more clear separation of the contexts, while our further selection procedure (described below) ensured that items were included across a wide range of this ranked continuum.

After creating the ranked continuum of items, inclusion moved from highest to lowest ranked items. Each of the 31 items, as described in Appendix 2, was included a minimum of three times and maximum of four times across the entire selection, performed by different actors, while ensuring that each item also appeared at least once in more-communicative context and once in the less-communicative context. Three videos from each actor in the previous study were included. This ensured an even representation of the data on which we previously reported. Supplementary Figure 1 illustrates the range of kinematics, gaze, and video durations included across the two groups in the current study with respect to the original dataset.

We ensured that the current stimulus set was representative of the original data by repeating the same mixed model analyses described in Trujillo, Simanova et al. (2018). In line with the original dataset, we found significantly higher values in communicative compared to non-communicative vertical amplitude (communicative = 0.160 ± 0.99; non-communicative = − 0.449 ± 0.809; *χ* ^2^(4) = 12.263, *p *< 0.001), submovements (communicative = 0.161 ± 789; non-communicative = − 0.661 ± 585; *χ* ^2^(4) = 32.821, *p *< 0.001), peak velocity (communicative = 0.181 ± 1.08; non-communicative = − 0.683 ± 0.649; *χ* ^2^(4) = 23.965, *p *= 0.001), and direct eye-gaze (communicative = 0.235 ± 0.220; non-communicative = 0.013 ± 0.041; *χ* ^2^(4) = 44.703, *p *< 0.001). Also in line with the original data, we found a less robust, but still significant difference in hold time (communicative = 0.107 ± 1.159; non-communicative = − 0.448 ± 0.892; *χ* ^2^(4) = 7.917, *p *= 0.005), Finally, duration was also longer in communicative (*M* = 7.237 ± 1.754) compared to non-communicative (*M* = 6.132 ± 1.235) videos.

## Appendix 2: List of items from Trujillo, Simanova et al. (2018)

The table provides the original Dutch response options that participants saw, alongside the English translation.

**Table:**

Original (Dutch)	English
appel verplaatsen	Move apple
banaan pellen	Peel banana
blokken stapelen	Stack blocks
brood snijden	Cut bread
citroen uitpersen	Squeeze lemon
dobbelstenen gooien	Roll dice
haar borstelen	Brush hair
hoed opdoen	Put on hat
kaarten schudden	Shuffle cards
kurk verdwijderen	Remove cork
naam schrijven	Write name
papier afvegen	Brush-off paper
papier knippen	Cut paper
papier kreukelen	Crumple paper
papier meten	Measure paper
papieren nieten	Staple papers
papier scheuren	Tear paper
papier stempelen	Stamp paper
papier vouwen	Fold paper
pendop opdoen	Put on pen cap
pendop verdwijderen	Remove pen cap
potje openmaken	Open jar
ring aandoen	Put on ring
slot openmaken	Open lock
spijkers slaan	Hammer nails
tafel schrobben	Scrub desk
tekening wissen	Erase drawing
thee roeren	Stir tea
theezakje dompelen	Steep tea
water gieten	Pour water
zonnebril opdoen	Put on sunglasses

## Appendix 3: Mixed effects modeling procedure

The order in which the predictor variables were entered into the mixed effects model was determined based on the a priori hypothesized contribution of the three components: range of motion has been found to be increased in adult–child interactions (Brand et al., 2002; Fukuyama et al., 2015); peak velocity was found to be increased in a communicative context in at least one study (Trujillo, Simanova et al., 2018); punctuality was previously not found to be changed in child–adult interactions by (Brand et al., 2002), but was found to be increased in a communicative context by (Trujillo, Simanova et al., 2018).

As more-communicative videos were, on average, longer than less-communicative videos, we included video duration (ms) in our regression models. This allowed us to test the contribution of kinematic features after taking into account total duration, ensuring that any effect of kinematics is not explained by duration alone. We report the video duration correlation from the best-fit model if this model is a better fit to the data than the null model. If the null model is a better fit, then we report the video duration correlation from the null model. Duration was fitted before the kinematic variables in order to ensure that any significant contribution of kinematic modulation to the model fit was over and above that of duration. In other words, our models were set up to specifically test the contribution of kinematic modulation after taking into account video duration and inter-individual differences.

Typically, when utilizing mixed effects models the researcher must first find the model that is the best-fit for the data before making inferences on the model parameters. The best-fit model was determined by first defining a ‘null’ model that only included duration as fixed effect and participant as random intercept. We used a series of log-likelihood ratio tests to determine if each kinematic feature term (described above: range of motion, velocity, punctuality) contributed significantly to the model fit. For example, if a comparison between a model that includes peak velocity and a model that does not include this effect term yields a non-significant result, then we do not include this kinematic feature in the model. If the comparison yields as a significant result, we keep this kinematic feature and compare this model with a new model that contains the next non-tested kinematic feature. In a step-wise fashion we thus test the contribution of each of the kinematic features. We report effects from the final, best-fit model, if it is still a better fit than the null model.
