# Supplementary material for: The communicative advantage: how kinematic signaling supports semantic comprehension
Source: Psychol Res. 2019 May 11;84(7):1897–911. doi: 10.1007/s00426-019-01198-y (PMC7772160; doi:10.1007/s00426-019-01198-y)
Supplement: Supplementary file 5 — Supplementary material 5 (DOCX 1830 kb) [file 426_2019_1198_MOESM5_ESM.docx]

**Supplementary Material**


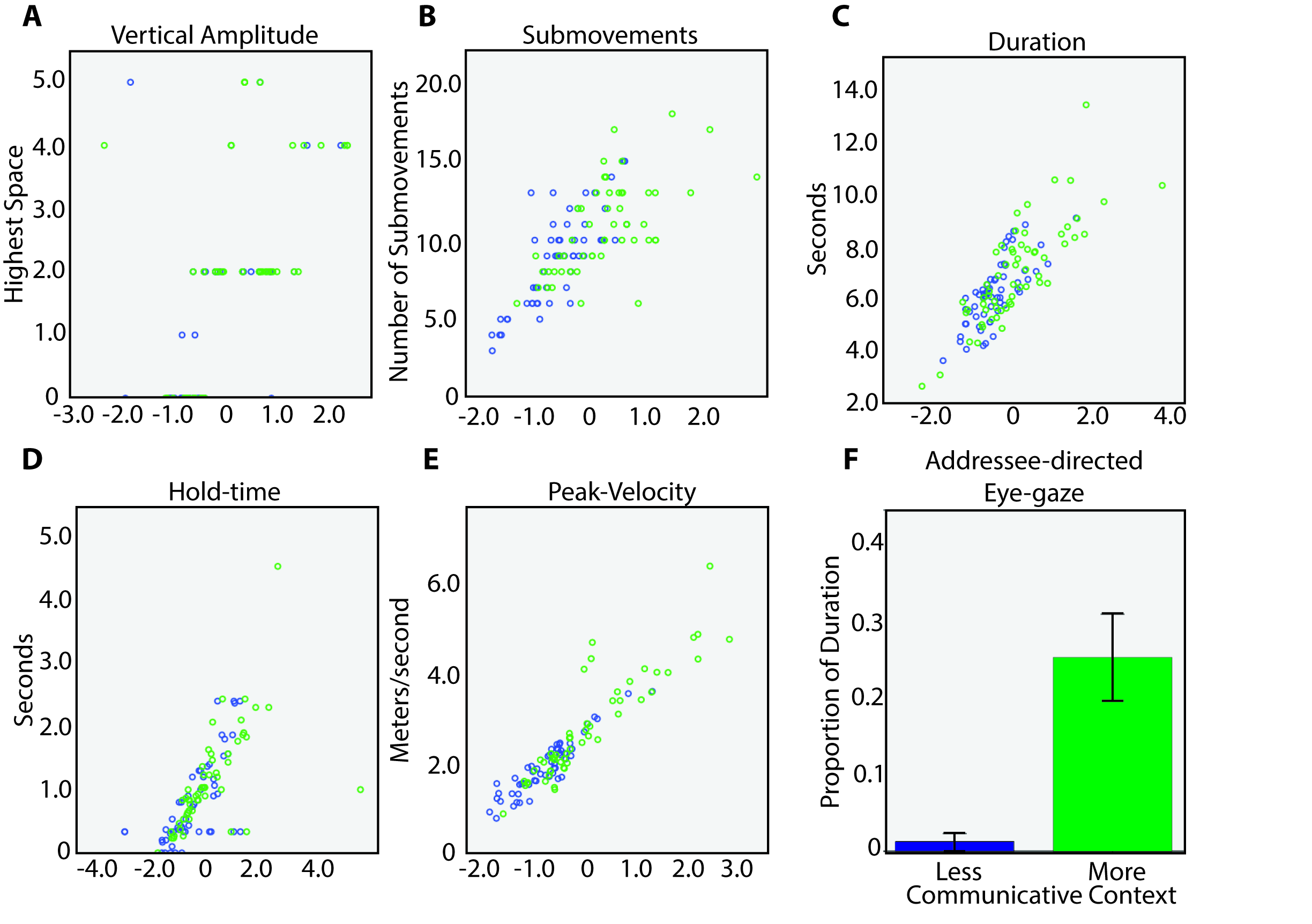


Supplementary Figure 1. **A-E**. Overview of raw and modulation values for kinematics and duration of included videos. In all scatter plots the y-axis depicts raw values, while the x-axis depicts modulation (z-score) values. Blue circles are less-communicative videos, green circles are more-communicative videos. **F**. Comparison between more-communicative and less-communicative selections of the proportion of the total duration during which addressee-directed eye-gaze was detected.

Supplementary Table 1. Comparison of video durations across conditions for Experiment I

|  | **df** | ***F*** | ***p*** |
| --- | --- | --- | --- |
|  |  | **Initial** |  |
| **Face visibility** | 1 | 0.001 | 0.977 |
| **Communicative Context** | 1 | 0.202 | 0.656 |
| **Residual** | 34 |  |  |
|  |  | **Medium** |  |
| **Face visibility** | 1 | 0.642 | 0.429 |
| **Communicative Context** | 1 | 3.404 | 0.074 |
| **Residual** | 34 |  |  |
|  |  | **Final** |  |
| **Face visibility** | 1 | 2.361 | 0.133 |
| **Communicative Context** | 1 | 3.129 | 0.086 |
| **Residual** | 34 |  |  |
